# Silver Sutures

**Published:** 1860-01

**Authors:** 


3. Silver Sutures.—On the 17th Dec. last, we had occasion to excise an extension cicatrix, the result of a burn on the popliteal region. The loss of substance was so extensive as to require moderate traction to fetch the edges of the wound together. In order to test the comparative value of sutures of silver wire and of thread, a part of the wound was dressed with the former, and a part with the latter. On the second day, nearly all the wires had cut through, notwithstanding strips of adhesive plaster were applied when separation of the edges was perceived. The thread, on the contrary, retained the edges well in contact to the fifth day.

This result was owing, no doubt, to the fineness of the wire used, which passes upon a smaller surface than that with which the thread comes in contact. We infer that if metalic sutures are to be used, for which, in general, no good reason appears as yet, those of lead or silver, made broad, should be preferred.

We have also recently used the silver wire on a stump, after amputation of the arm, at the City Hospital. The result was, however, in no respect different from that where thread is used.

